# Antioxidant and Anti-Inflammatory Activities of Phenolic Acid-Rich Extract from Hairy Roots of *Dracocephalum moldavica*

**DOI:** 10.3390/molecules28196759

**Published:** 2023-09-22

**Authors:** Izabela Weremczuk-Jeżyna, Weronika Gonciarz, Izabela Grzegorczyk-Karolak

**Affiliations:** 1 Department of Biology and Pharmaceutical Botany, Medical University of Lodz, Muszynskiego 1, 90-151 Lodz, Poland; izabela.weremczuk-jezyna@umed.lodz.pl; 2 Department of Pharmaceutical Microbiology and Microbiological Diagnostic, Medical University of Lodz, Banacha 12/16, 90-237 Lodz, Poland; weronika.gonciarz@biol.uni.lodz.pl

**Keywords:** *Dracocephalum moldavica*, hairy roots, plant extraction, rosmarinic acid, caffeic acid, salvianolic acid B

## Abstract

This study evaluates the antioxidant properties and anti-inflammatory potential of polyphenolic acid-rich fractions of 80% methanolic extract from the hairy roots of *Dracocephalum moldavica*. The fractionation of the crude extract yielded the following: a diethyl ether fraction rich in caffeic acid (DM1) (25.85 mg/g DWE), an n-butyl fraction rich in rosmarinic acid (DM3) (43.94 mg/g DWE) and a water residue rich in salvianolic acid B (DM4) (51.46 mg/g DWE). The content of these compounds was determined using high-performance liquid chromatography (HPLC). Their antioxidant activity was evaluated based on DPPH (2,2-diphenyl-1-picrylhydrazyl), ABTS (2,2′-azino-bis(3-ethylbenzothiazoline-6-sulphonic acid) diammonium salt) and FRAP assays. The anti-inflammatory activity of the fractions was determined by their effect on nuclear factor kappa-B (NF-κB) activation and interleukin-1*β* (IL-1*β*) production in LPS *E. coli* stimulated monocytes. The level of pro-inflammatory IL-1*β* in cells was measured using ELISA. The activation of NF-κB in THP1-Blue™ cells, resulting in the secretion of SEAP (secreted embryonic alkaline phosphatase), was detected spectrophotometrically using Quanti-Blue reagent. Among the tested fractions, the diethyl ether fraction (DM1) showed the highest antioxidant potential, with an EC_50_ value of 15.41 µg/mL in the DPPH assay and 11.47 µg/mL in ABTS and a reduction potential of 10.9 mM Fe(II)/g DWE in FRAP. DM1 at a concentration of 10 mg/mL also efficiently reduced LPS-induced SEAP secretion (53% inhibition) and IL-1*β* production (47% inhibition) without affecting the normal growth of L929 fibroblast cells.

## 1. Introduction

The *Dracocephalum* genus of the Lamiaceae family contains over 70 species, which are mainly widespread in Northern Hemisphere regions [1]. One of the most widely known of this genus is *D. moldavica*, an aromatic plant with medicinal uses. Although the species is native to central areas of Asia, it also occurs in central and eastern European regions [2]. The aerial parts of *D. moldavica* are used in traditional medicine as remedies in hypertension, cardiac disease, atherosclerosis or asthma [3]. Their pharmacological activity is related mainly to the presence of bioactive phenolic acids and flavonoids. Among them, the most important seem to be rosmarinic acid, caffeic acid, apigenin, quercetin, acacetin, luteolin and their derivatives [2].

Polyphenols are able to modify the expression of genes encoding pro-inflammatory enzymes such as cytokines, lipoxygenase and cyclooxygenase; this, combined with their antioxidant potential, such as reactive oxygen species (ROS) scavenging, allows them to regulate inflammatory signaling. Their mechanism of action mainly involves the TNF-α/NFκB signaling pathway [4]. The anti-inflammatory effect of phenolic acids present in *D. moldavica* and their potential use as therapeutic agents in acute or chronic disorders have been confirmed in some in vitro and in vivo studies [4].

However, to fully explore the potential of polyphenol-rich plant material in, for example, dietary supplements or pharmaceuticals, a method for effective selective extraction is necessary. Due to the complexity and interaction of phenolic acids with other compounds, there is no single, well-defined method or solvent suitable for their extraction. However, it is widely believed that the most effective approach uses a mixture of water and alcohol [5]. The addition of a small amount of water to the organic solvent usually creates a more polar environment that increases the diffusion of polyphenols from the plant material [5]. Such water-alcoholic extracts are usually rich in a range of phenolic compounds; however, they additionally contain undesirable substances such as pigments, waxes or sugars and others and therefore require additional purification.

One method for removing ballast substances from the extract and pre-separating phenolic compounds is liquid–liquid extraction (LLE). This method is based on the principle that the compounds can be distributed in a certain ratio between two immiscible solvents, usually the aqueous phase and the organic phase (diethyl ether, ethyl acetate, n-butanol). Solvents with different polarities are needed to dissolve bioactive phenolic compounds of similar polarizations [6]. The use of LLE allows relatively pure and concentrated fractions to be obtained and, thus, a plant extract with greater biological activity compared to the crude extract.

Our previous studies indicate that the hairy roots of *D. moldavica* are an excellent source of phenolic acids. The crude hydromethanolic extract (2:8 *v*/*v*) from this plant material demonstrated a high content (78 mg/g DW) of rosmarinic acid [7]. The current work is a continuation of this research. Its aim was to obtain concentrated fractions of phenolic acids from hairy roots of *D. moldavica* using the LLE method, determine their bioactive compound content and evaluate their biological potential. The obtained fractions were evaluated for antioxidant properties using a FRAP assay, based on iron ion reduction, and “scavenging” DPPH and ABTS radical assays. The study also examines their anti-inflammatory effects: their ability to inhibit NF-κB, activation and pro-inflammatory IL-1*β* secretion by human monocytes treated with *E. coli* LPS.

## 2. Results

### 2.1. Determination of Phenolic Acids Content

Lyophilized hairy roots of *D. moldavica* were extracted with a methanol:water (8:2 *v*/*v*) solution: the extraction yield amounted to 35.6% ± 2.1. Next, the dried crude hydromethanolic extract was dissolved in water and fractionated sequentially using the following organic solvents: diethyl ether (DM1), ethyl acetate (DM2) and n-butanol (DM3) with respective extraction yields of 3.6% ± 0.07, 4.4% ± 0.05 and 37.1% ± 0.98; in addition, the water residue (DM4) yielded 36.7% ± 0.76.

**Figure 1:**
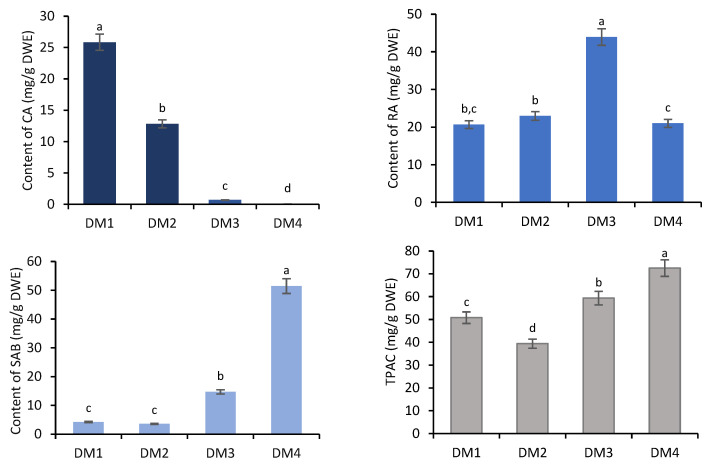
Accumulation of caffeic acid (CA), rosmarinic acid (RA) and salvianolic acid B (SAB) and their total content (TPAC) in diethyl ether (DM1), ethyl acetate (DM2) and n-butanol (DM3) extracts and water residue (DM4) obtained from hairy roots of *D. moldavica.* The values are given as the means ± SD of three independent experimental replicates. The means marked with various letters for the same metabolite differ significantly at *p* < 0.05 according to Tukey’s test.

All of the obtained fractions were quantitatively analyzed by high-performance liquid chromatography (HPLC). The fractions DM1–DM4 contained salvianolic acid B (SAB), rosmarinic acid (RA) and caffeic acid (CA). The total phenolic acid content (TPAC) in individual fractions ranged from 39.41 to 72.51 mg/g dry weight of extract (DWE), with the highest total content recorded for DM4 (Figure 1).

The dominant component of DM4 was SAB (51.46 mg/g DWE; >70% of TPAC) (Figure 1). Much lower SAB levels were found in the fractions DM1–DM3, ranging from 3.60 (8%) to 14.74 mg/g DWE (25%). RA was the predominant component of both the n-butanol (DM3; 43.94 mg/g DWE) and ethyl acetate fractions (DM2; 22.96 mg/g DWE). Although a similar RA level as in the DM2 was found in the least polar fraction (DM1) (20.69 mg/g DWE), this was dominated by caffeic acid (25.85 mg/g DWE), whose content decreased in the sequence: DM1 > DM2 > DM3 > DM4 (Figure 1).

### 2.2. Biological Activity of D. moldavica Hairy Roots

#### 2.2.1. Antioxidant Activity

The antioxidant potential (EC_50_) of fractions of *D. moldavica* extract, determined by DPPH, ranged from 15.41 to 42.61 µg/mL (Table 1). The highest anti-DPPH activity was observed for the diethyl ether fraction (EC_50_ = 15.41 μg/mL). Also, the ethyl acetate fraction (DM2) had a higher ability to scavenge radicals (23.53 μg/mL) than the synthetic antioxidant butylhydroxytoluene (BHT) (EC_50_ = 26.40 μg/mL), although not as strong as that of the vitamin E derivative, Trolox (4.72 μg/mL) (Table 1). The ABTS assay found the tested fractions to have strong anti-radical activity, with EC_50_ values ranging from 11.47 to 24.20 µg/mL; moreover, no significant difference in activity was found between the strongest fraction, DM1, and BHT (*p* < 0.05) (Table 1). The strongest reduction properties (FRAP) were observed for DM2 (12.86 mM Fe(II)/g DWE), followed by DM1 (Table 1). The DM3 fraction was half as strong, although it still exhibited a greater reduction potential than the controls. The weakest reduction potential was shown by the DM4 fraction.

Our studies indicated that the DM1 fraction demonstrated the highest antioxidant potential, which is probably related to its high caffeic acid content (Figure 1). This is confirmed by the strong or very strong correlation between the CA content and the results of the antioxidant assays DPPH (r = −0.971), ABTS (r = −0.875) and FRAP (r = 0.762) (Table 2).

#### 2.2.2. Cytotoxicity and Anti-Inflammatory Activity

**Figure 2:**
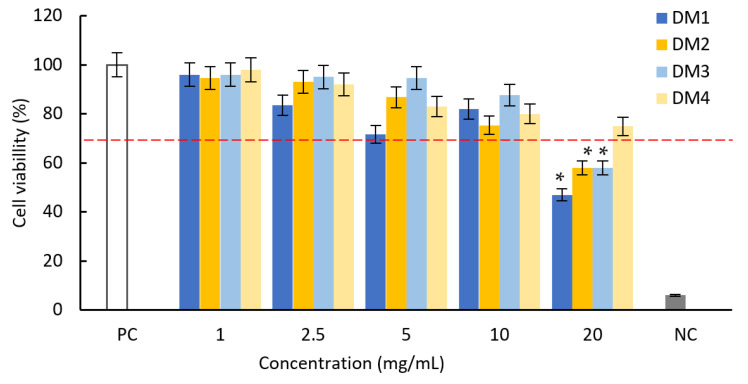
The influence of fractions DM1, DM2, DM3 and DM4 on L929 fibroblast viability based on the MTT reduction assay. Results are shown as the mean ± standard deviation (SD). Three experiments were performed in triplicate for each experimental variant. Statistical analysis was performed using the Wilcoxon test, with significance at *p* < 0.05 (* unstimulated cells vs. extracts stimulated cells). A cell culture in a complete medium was used as the positive control (PC, 100% live cells); cells treated with H_2_O_2_ were used as negative controls (NC, 100% dead cells). The red line indicates the minimal percentage of viable cells required to confirm the extracts as non-cytotoxic in vitro.

The cytotoxicity of all tested fractions against L929 mouse fibroblasts was tested against L929 mouse fibroblasts using the MTT reduction test (Figure 2).

According to the ISO norm, the *D. moldavica* fractions within the concentration range 1–10 mg/mL did not show cytotoxic activity against mouse fibroblasts: the treatment resulted in a less than 30% reduction in cell viability. At the highest concentration (20 mg/mL), the cell viability decreased by 53% (DM1) and 42% (DM2 and DM3). These non-cytotoxic concentrations were then used in later studies to evaluate the effects of the extracts on the modulating activation of the NF-κB signaling pathway and the secretion of pro-inflammatory IL-1*β*.

**Figure 3:**
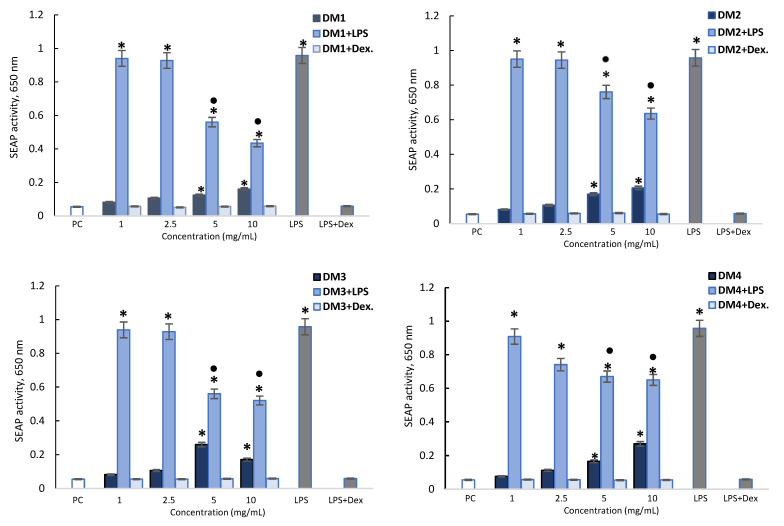
Activation of embryonic alkaline phosphatase (SEAP) in THP-1XBlue monocytes. THP-1XBlue monocytes were stimulated with lipopolysaccharide (LPS) *E. coli* alone (negative control) and *D. moldavica* fractions (DM1-DM4) alone or in combination with LPS *E. coli* (1 µg/mL) or in combination with dexamethasone (Dex.). Cell cultures in a complete medium were used as positive controls (PC). The experiments were performed three times in triplicate for each experimental variant. The results are shown as the mean ± standard deviation (SD). Statistical analysis was performed using the nonparametric Wilcoxon test (*p* < 0.05) (* unstimulated cells vs. stimulated cells; ● LPS vs. LPS + *D. moldavica* fraction).

In the experiment, the cell culture supernatants were collected and subjected to the spectrometric assessment of the secreted embryonic alkaline phosphatase (SEAP) level. *E. coli* THP-1XBlue monocytes stimulated with lipopolysaccharide (LPS) were found to produce a high level of SEAP, indicating the activation of nuclear transcription factor NF-κB. This SEAP activation was abrogated by dexamethasone (Figure 3). The *D. moldavica* fractions alone at the higher concentration (5 and 10 mg/mL) also induced higher levels of SEAP than the control. However, all DM fractions (5 and 10 mg/mL) significantly diminished SEAP stimulation by LPS *E. coli*. The DM1 and DM3 fractions had the strongest effect, reducing SEAP activity by 41.5 to 54.5%, compared to DM2 and DM4, which reduced the activity by 23.7 to 33.6% (Figure 3).

**Figure 4:**
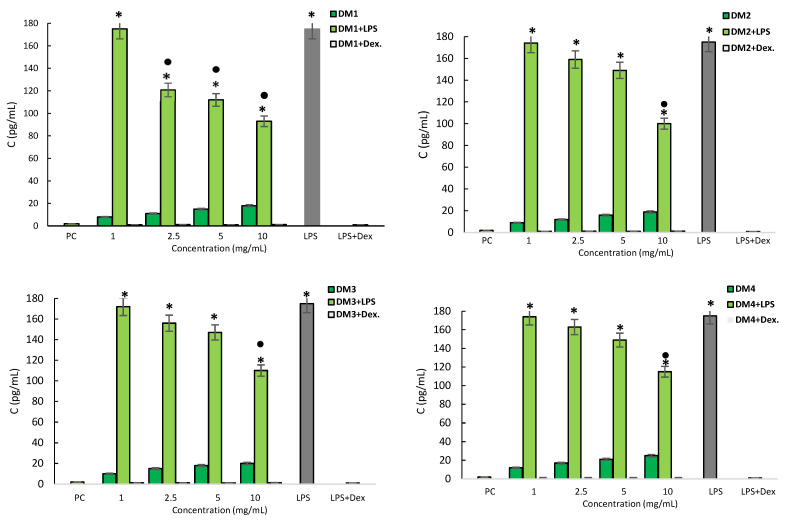
Production of pro-inflammatory IL-1*β* by THP-1 monocytes. THP-1 monocytes were stimulated with lipopolysaccharide (LPS) *E. coli* alone (negative control) and *D. moldavica* fractions (DM1-DM4) extract alone or in combination with LPS *E. coli* (1 µg/mL) or in combination with dexamethasone. Cell cultures in a complete medium were used as positive controls (PC). The results are presented as the mean values ± standard deviation (SD). Three experiments were performed in triplicate for each experimental variant. Statistical analysis was performed using the nonparametric Wilcoxon test (*p* < 0.05) (* unstimulated cells vs. stimulated cells; ● LPS vs. LPS+ *D. moldavica* fraction).

The monocyte supernatant interleukin level was assessed by an enzyme-linked immunosorbent assay (ELISA). LPS was found to cause a significant increase in IL-1*β* secretion by monocytes, and this production was fully halted by dexamethasone (Figure 4). THP-1 monocytes stimulated with *D. moldavica* fractions, at any concentration, did not respond to increased IL-1*β* production. In addition, all DM fractions decreased IL-1*β* production following LPS treatment in a dose-dependent manner. The strongest effect was found for the DM1 fraction, which reduced IL-1*β* secretion by over 30% at 2.5 mg/mL; however, all fractions significantly reduced the interleukin level by 35 to 47% at a concentration of 10 mg/mL (Figure 4).

## 3. Discussion

Extracts obtained from medicinal plants are an excellent source of secondary metabolites with antimicrobial, antioxidant and anti-inflammatory activities, which have been found to be effective against cancer, diabetes and heart arrhythmia. They can also serve as relatively inexpensive ways to prevent civilization disease [8]. To fully exploit the therapeutic potential and pharmacological properties of plant extracts and their secondary metabolites, it is important to not only identify their bioactive compounds but also to find the optimal extraction method. One of the important factors in the preparation of extracts is the use of selective solvents, as this allows high extraction yields to be obtained for individual components [9]. 

In the present study, the starting point for obtaining the tested fractions from the dried hairy roots of *D. moldavica* was extraction with 80% methanol. This solvent was previously indicated as a suitable extractor of phenolic compounds from an in vitro culture of *Dracocephalum* spp. [10,11]. In this condition, a higher extraction yield (35.6%) was obtained compared to that in other members of the Lamiaceae, e.g., 18.8% from the roots of *Salvia bulleyana* and 23.6% from the aerial parts, and 31.7% from the leaves of *Origanum campatum* [12,13]. 

The next step is to remove the ballast substances, i.e., the mixture of phenolic and non-phenolic substances such as sugar, fatty acids, organic acids and wax, present in the crude extract and pre-separate the phenolic compounds. A suitable method is liquid–liquid extraction (LLE), which relies heavily on the solubility of phenolic compounds in certain organic solvents. LLE yields relatively pure samples with high concentrations of metabolites [14]. 

According to several reports, the diffusion of phenolic compounds from the inorganic solvent to organic solvents of different polarities depends on the number of hydroxyl groups attached directly to an aromatic ring, the size of molecules and their stereochemistry [15]. The diffusion coefficient and rate of dissolution of compounds until they reach an equilibrium concentration inside the solvent are also important [16]. Galanakis et al. [17] report that some phenolic acids, e.g., caffeic acid, can be effectively recovered with diethyl ether and ethyl acetate. This compound has only two -OH groups and demonstrates greater affinity to less polar solvents compared to rosmarinic acid, with four -OH groups. Additionally, RA, with two anti-diametric substituted carbons, prefers an alcohol-based solvent [17]. In the present study, the use of LLE allowed for the partial separation of the compounds contained in the hydromethanolic extract, with CA dominating in the least polar fraction and SAB in the most polar fraction. 

Due to their promising properties, phenolic acids such as CA, RA and SAB have been the subject of many studies in the last 20 years [18,19,20]. These bioactive compounds are characteristic of popular aromatic plant species from the Lamiaceae family (*Salvia* spp., *Origanum* spp., *Lavandula* spp.), and were also described in the genus *Dracocephalum* [21,22]. The compounds are of great interest due to their low toxicity and high safety, as well as their varied bioactivity profile, including their anti-inflammatory and antioxidant effects [23].

The antioxidant potential of the polyphenolic acid-rich fractions obtained from the tested hydromethanolic extract was assessed using spectrophotometric methods. More precisely, the study used the FRAP, DPPH and ABTS assays, which are commonly used for studying plant extracts [24]. The FRAP assay is based on single electron transfer and determines the ability of antioxidants to reduce ferric ion (Fe^3+^) to ferrous ion (Fe^2+^) in an iron salt complex [Fe^3+^–(TPTZ)_2_]^3+^, while the DPPH and ABTS assays are mainly based on free radical scavenging [25]. In all cases, the reaction is associated with a change in the color of the solution. In products of plant origin, the antioxidant properties measured by these methods are usually proportionate to the concentration of polyphenolic compounds. 

In the DPPH assay, the DM1 fractions demonstrated a lower EC_50_ value than the crude hydromethanolic extract of hairy roots of *D. moldavica* (EC_50_ = 26.56 μg/mL) and the roots of field-grown plants (EC_50_ = 44.62 μg/mL) [7]. The ethyl acetate fraction of *D. moldavica* (DM2) was comparable to the ethyl acetate extract from *D. multicaule* aerial parts (EC_50_ = 25.00 μg/mL) [26]. Both of the most potent fractions (DM1 and DM2) showed a stronger ability to neutralize the DPPH radical than the control synthetic antioxidant BHT. They were also more active than those noted in several other studies on *Dracocephalum* spp. For example, the methanolic extract from aerial parts of *D. heterophyllum* scavenged DPPH radicals with an EC_50_ at 37.0 μg/mL [27], whereas ethyl acetate extracts of *D. multicaule*, *D. kotschyi* and *D. polyachetum* were found to have EC_50_ values of 25.0, 47.2 and 50.5 μg/mL, respectively [26]. In contrast, Olennikov et al. [22] found the ethanolic extract of the aerial parts of wild *D. palmatum* to have slightly stronger antioxidant potential, with EC_50_ values of 17.73 μg/mL (DPPH) and 6.35 μg/mL (ABTS) and a reduction potential of 12.22 mM Fe(II)/g (FRAP). The strong activity of *D. palmatum* may be related to the accumulation of a significant number of flavonoids in shoots, in addition to phenolic acids.

Our findings indicate that the strong antioxidant potential of *D. moldavica* hairy roots is mainly due to the presence of CA (DM1 fraction), followed by RA and SAB. CA also significantly contributes to the antioxidant activity of other Lamiaceae members such as *Lavendula angustifolia* [28], *Mentha spicata* [29] or *Salvia* spp. [30]. CA owes its strong antioxidant potential to the presence of a catechol moiety and two hydroxy groups attached to the aromatic ring. The catechol moiety allows for the stabilization of a phenoxy radical intermediate through intramolecular hydrogen bonding [31], while the hydroxyl groups react with free hydroxyl radicals and superoxide anions [32].

The number of hydroxyl groups in the aromatic ring usually positively correlates with the antioxidant activity [31,32]. However, our findings indicate that the fractions DM3 and DM4 of *D. moldavica* extract containing high levels of RA and SAB, whose molecules contain more hydroxyl groups than CA, possess a lower antioxidant capacity than DM1 (Table 1). On the other hand, some research revealed that although hydroxyl substituents have a strong influence on antioxidant capacity, this can be modified by many other factors, such as the configuration of hydroxyl groups, their availability, the stereochemistry of the molecule and its size [33,34]. In addition, it should be emphasized that despite being weaker than DM1 and DM2, the DM3 and DM4 fractions also showed strong antioxidant properties in all tests; this is little surprise considering that their main ingredients, RA and SAB, have proven antioxidant activities in vitro and in vivo. For example, previous studies indicate that SAB and RA effectively scavenged DPPH with IC_50_ values of 2.20 ± 0.30 µg/mL and 1.06 ± 0.13 µg/mL, respectively [35,36].

The anti-inflammatory effects of fractions obtained from 80% methanolic extract of *D. moldavica* were evaluated in a cell-based assay using human monocyte THP-1 cells, which are widely used to study the modulation of pro-inflammatory effects. Stimulation by the pro-inflammatory factor LPS *E. coli* induced the production of pro-inflammatory cytokines, such as IL-1*β* and activated NF-κB, in the monocytes. NF-κB is a complex protein that plays a key role in transcription, cytokine production and cell survival. It controls the immunity, inflammation, stress, proliferation and apoptotic responses of a cell in multiple ways [37]. Upon LPS stimulation, activated NF-κB promoted the secretion of SEAP in THP-1XBlue cells. 

Our findings demonstrate that the different fractions obtained from *D. moldavica* hairy roots were able to downregulate the production of IL-1*β* and significantly diminish the level of SEAP produced by LPS *E. coli* stimulated macrophages. Of these, DM1, which is rich in CA, exhibited the highest activity. Several studies have confirmed the protective effect of CA against LPS-induced damage. For example, Liu et al. [38] report that 50 μg/mL CA significantly inhibited the LPS-induced NF-κB activation of bovine mammary epithelial cells. The effect of CA was at least partly achieved by reducing the production of proinflammatory cytokines (IL-8, IL-1β, IL-6) and the tumor necrosis factor α [38].

Previous studies have demonstrated that rich-in-RA methanolic extract from the aerial part of *Salvia officinalis* can inhibit NF-κB production in LPS-induced RAW 264.7 cells with IC_50_ values of 50 μg/mL, thus playing an important role in controlling inflammatory processes associated with cancer progression [39]. Similarly, extract from *Perilla frutescens* attenuated lipopolysaccharide-induced inflammation in RAW 264.7 cells by inhibiting the expression of TNF-*α*, IL-1*β* and IL-6 via the suppression of NF-κB activation and translocation [40]. In addition, it was shown that 1.25 mg/mL shoot or root extract of *Salvia cadmica* reduced NF-κB pathway activation and pro-inflammatory IL-1*β* and TNF production in human THP1 macrophages treated with *E. coli* or *Helicobacter pylori* LPS [41]. In all the above studies, as in the case of *D. moldavica*, polyphenolic acids were identified as the main metabolites, including CA, RA and SAB.

Several studies have shown that phenolic acids such as CA, RA and SAB can inhibit the expression of pro-inflammatory factors such as TNF-α, IL-1*β* and IL-6 to exert an anti-inflammatory effect [42,43,44]. Taïlé et al. [45] found that 10 μM CA exerted anti-inflammatory action activity by reducing IL-6 secretion by about 50%. Liu et al. [46] report that 25 μM SAB decreased the secretion of IL-1*β*, IL-6 and TNF-*α* by, respectively, 57.0%, 60.4% and 48.3%; in addition, RA inhibited IL-1*β*, IL-6 and TNF-*α* by 39.4%, 37.1% and 38%, respectively. 

Liu et al. (2018) suggest that the anti-inflammatory properties of phenolic acids from the roots of *S. miltiorrhiza* involve the TLR-4/NF-κB signaling pathway. Polotrak et al. [47] report that TLR4 is a critical receptor for NF-κB whose increased activity is associated with progressive inflammation [37]. In unstimulated cells, NF-κB dimers located in the cytoplasm could bind to the IκB repressor by noncovalent binding [48]. After the phosphorylation of IκB and its subsequent degradation, free NF-κB is moved into the nucleus to regulate the production of inflammatory genes [49,50].

## 4. Materials and Methods

### 4.1. Plant Material and Preparation of Extracts

Hairy roots of *D. moldavica* were induced using *Agrobacterium rhizogenes* (strain A4) and cultivated in dark in a half-strength B5 liquid medium (½B5) [7]. In the present study, 5 g of roots (air-dried under room temperature) were ground into a fine powder using a mortar and pestle and extracted with chloroform (50 mL) using a UD-20 ultrasonic disintegrator (ChemLand, Stargard, Poland). After filtration, the samples were extracted three times with 80% aqueous methanol (50 mL, 15 min) at 40 °C using the ultrasonic disintegrator. The obtained extracts were combined and evaporated to dryness under reduced pressure. The dried crude hydromethanolic extract (1.84 g) was suspended in water (200 mL) and subjected to liquid–liquid extraction with organic solvents in the following sequence: diethyl ether (DM1), ethyl acetate (DM2) and n-butanol (DM3). The crude extract in water was mixed with 100 mL of an appropriate solvent in a separation funnel (300 mL) and shaken for 20 min; following this, the organic solvent was removed. This procedure was repeated five times for each solvent. The solvent fractions were combined and evaporated to dryness in a vacuum. This extraction process yielded the following fractions: DM1—0.063 g, DM2—0.077 g, DM3—0.646 g and DM4 (water residue)—0.639 g.

### 4.2. Quantitative HPLC Analysis

For HPLC analysis, the samples (DM1–DM4) were dissolved in 10 mL of 80% (*v*/*v*) aqueous methanol and filtered through a PTFE syringe filter (25 mm, 0.22 µm). The phenolic acid contents were evaluated using an Agilent Technologies 1290 Infinity HPLC apparatus (Santa Clara, CA, USA) with a diode array detection (DAD) and Eclipse XDB-C_18_ column (150 × 4.6 mm, 5 µm). The mobile phase (A) was acetonitrile, and the mobile phase (B) was 0.1% formic acid in water. The solvent system used for elution was: 0–5 min, 10% A and 90%; 5–20 min, 18% A and 82% B; 20–25 min, 38% A and 62% B; 25–30 min, 100% A. The flow rate was 1.6 mL/min, at a temperature of 35 °C. The detection wavelength was set at 325 nm. Individual calibration curves for the reference standards CA, RA and SAB (Sigma-Aldrich, Darmstadt, Germany) were constructed. The following regression equations were formulated based on the peak areas: CA y =23.75389x − 20.3788 (r^2^ = 0.9639), RA y = 12.1228x + 1281.4666 (r^2^ = 0.98129) and SAB y = 3.5352x − 20.3788 (r^2^ = 0.99571). The amount of the compound in the plant material was expressed as mg/g DWE.

### 4.3. Antioxidant Assays

The antioxidant activity of the fractions (DM1–DM4) was evaluated using the DPPH free radical scavenging assay according to Grzegorczyk-Karolak and Kiss [51]. Samples (2 mL) at concentrations between 1 and 400 μg/mL were added to 2 mL of DPPH (0.2 mM) and incubated for 30 min in room temperature; following this, absorbance was read using a spectrophotometer (Ray-Leight, UV 1061, Beijing, China) at 517 nm. The results were expressed as the EC_50_ value (μg/mL), i.e., the concentration of the extract that reduces the initial radical concentration by 50%.

The antiradical activity of the fractions against ABTS was determined as described by Grzegorczyk-Karolak and Kiss [51]. Radical ABTS^•+^ was prepared through the oxidation of ABTS by potassium persulfate. For all fractions, samples at concentrations between 1 and 200 μg/mL were mixed with the same amount of ABTS solution and were incubated for 10 min at 25 °C. The decrease in absorbance was measured at 734 nm, and the results were expressed as the EC_50_ value (μg/mL).

The FRAP method is based on the reduction of the Fe^3+^-TPTZ complex to Fe^2+^-TPTZ. The antioxidant capacity of the fractions was estimated spectrophotometrically according to Grzegorczyk-Karolak and Kiss [51]. Samples containing fractions, water and FRAP reagent were incubated at 37 °C for 15 min. Following this, absorbance of the solutions was measured at 595 nm. The results were expressed in mM Fe (II)/g DWE.

BHT, a synthetic antioxidant, and Trolox, a derivative of vitamin E purchased from Sigma–Aldrich (Darmstadt, Germany), were used as positive controls in antioxidant assays.

### 4.4. Cell Culture

Reference L929 mouse fibroblasts (LGC Standards, Middlesex, UK) and the human THP-1 line (ATCC^®^ TIB-202 ™) were cultured in Roswell Park Memorial Institute-1640 medium supplemented with 10% heat-inactivated fetal bovine serum and the standard antibiotics penicillin (100 U/mL) and streptomycin (100 µg/mL) in a humid atmosphere containing 5% CO_2_ and 95% air at 37 °C. All cell culture components were obtained from Biowest, Nuaillé, France. Human THP-1XBlue Cells (Invitrogen, San Diego, CA, USA) were used to assess nuclear factor kappa B (NF-κB). THP1-Blue™ cells were derived from the human THP-1 monocyte cell line by the stable integration of an NF-κB-inducible embryonic alkaline phosphatase reporter construct. The activation of NF-κB resulted in the secretion of SEAP, which could be detected spectrophotometrically using Quanti-Blue reagent (Invitrogen, San Diego, CA, USA). The activation of the NF-κB signaling pathway was estimated as previously described [52]. The cells were passaged every three days to maintain a cell density < 2 × 10^6^ cells/mL.

### 4.5. Cell Stimulation Condition

THP-1XBlue Cells and THP-1 human monocytes were treated for 24 h with DM1, DM2 and DM3 or DM4 extracts at 1, 2.5, 5 or 10 mg/mL; some were exposed to extracts in combination with LPS *E. coli* (1 µg/mL) or with extracts in combination with dexamethasone (5 mg/mL). Cells were also treated with LPS of *E. coli* alone at a concentration of 1 µg/mL (negative control). 

After all treatments, the activation of the NF-kB transcription factor was assessed as described above, whereas the cell culture supernatant was collected, and the production of anti-inflammatory IL-1*β* was assessed by a commercial ELISA test (ThermoFisher, Waltham, MA, USA) with a sensitivity of 1 pg/mL [52]. Three independent experiments were performed in triplicate for each treatment.

### 4.6. MTT Reduction Assay

The cytotoxicity of the fractions (DM1–DM4) was tested using the L929 cell line (density of 2 × 10^5^ cells/mL) according to the ISO norm 10993–5 [53], as previously described [54]. The cell viability was estimated as the percentage of cells that were able to reduce tetrazolium salt (3-(4,5-Dimethylthiazol-2-yl)-2,5-Diphenyltetrazolium Bromide) (MTT). The effectiveness of MTT reduction was calculated by comparing the absorbance of treated cells to that of untreated control cells.

### 4.7. Statistical Analysis

Data are presented as the mean values ± SD. Means, standard deviations, IC_50_ values and correlation coefficients were calculated using the Microsoft Excel 2013 software (Microsoft Inc., Redmond, WA, USA). Statistical significance was accepted at a *p*-value < 0.05 using Wilcoxon or Tukey’s tests (STATISTICA 13 PL, StatSoft, Krakow, Poland).

## 5. Conclusions

It is necessary to optimize the extraction method to ensure the effective recovery of secondary metabolites from plant material. The fractionation of crude hydromethanolic extract from hairy roots of *D. moldavica* with solvents of suitable polarity obtained high levels of caffeic acid in the diethyl ether fraction (25.85 mg/g DWE), rosmarinic acid in the n-butyl fraction (43.94 mg/g DWE) and salvianolic acid B in the water residue (51.46 mg/g DWE). The tested fractions demonstrated high antioxidant activity, expressed as the antiradical and reduction potential, with the strongest potential demonstrated by the fraction dominated by caffeic acid. Furthermore, it appears that the mixture of polyphenols present in the *D. moldavicum* extract may modulate the inflammatory signaling pathways in differentiated THP-1 monocytes. Following treatment with the polyphenol-rich fractions, the monocytes exhibited downregulated IL-1*β* production and significantly diminished NF-κB activation that had been stimulated by LPS. However, the cellular response varied with the extract content; among the analyzed fractions, DM1 showed the best anti-inflammatory activity. Therefore, further studies are warranted to elucidate the mechanisms of the anti-inflammatory activity of bioactive compounds present in *D. moldavica* hairy roots.

In summary, purified concentrated fractions of 80% methanolic extract of *D. moldavica* roots are able to neutralize free radicals and demonstrate anti-inflammatory properties. These can support the body’s natural defense mechanisms and protect its cells from oxidation and the destructive effects of free radicals. As such, this material may be of considerable value in the prevention and treatment of civilization diseases.

## Figures and Tables

**Table 1:** Antioxidant activity of the extracts of *D. moldavica* hairy roots.

	DPPH EC_50_ (µg/mL)	ABTS EC_50_ (µg/mL)	FRAP (mM Fe (II)/g DWE)
DM1	15.41 ± 0.39 ^b^	11.54 ± 1.72 ^bc^	10.90 ± 0.81 ^b^
DM2	23.53 ± 1.03 ^c^	11.47 ± 0.65 ^c^	12.86 ± 0.24 ^a^
DM3	42.61 ± 1.37 ^e^	20.28 ± 1.1 ^d^	6.22 ± 0.13 ^c^
DM4	38.62 ± 1.63 ^e^	24.20 ± 2.08 ^d^	1.75 ± 0.04 ^f^
BHT	26.40 ± 1.26 ^d^	9.84 ± 0.85 ^b^	3.67 ± 0.27 ^e^
Trolox	4.72 ± 0.03 ^a^	4.44 ± 0.03 ^a^	5.23 ± 0.10 ^d^

The results are expressed as the means of three replicates ± SD. Values marked with the same letter are not significantly different at *p* < 0.05, according to Tukey’s multiple range test.

**Table 2:** Correlation coefficient (r) between the phenolic acid content in obtained fractions of *D. moldavica* extract and their antioxidant activity, evaluated using DPPH, ABTS and FRAP assays.

	CA	RA	SAB	TPAC
DPPH	−0.971	0.651	0.627	0.689
ABTS	−0.875	0.321	0.889	0.931
FRAP	0.762	−0.182	−0.930	−0.984

CA—caffeic acid, RA—rosmarinic acid, SAB—salvianolic acid B, TPAC—total phenolic acid content.

## Data Availability

The data are contained within the article.

## Sample Availability

Samples of the compounds are not available from the authors.
